# Computer-aided Discovery of Peptides that Specifically Attack Bacterial Biofilms

**DOI:** 10.1038/s41598-018-19669-4

**Published:** 2018-01-30

**Authors:** Evan F. Haney, Yoan Brito-Sánchez, Michael J. Trimble, Sarah C. Mansour, Artem Cherkasov, Robert E. W. Hancock

**Affiliations:** 1 Centre for Microbial Diseases and Immunity Research, University of British Columbia, Vancouver, British Columbia V6T1Z4 Canada; 2 Vancouver Prostate Centre, University of British Columbia, Vancouver, British Columbia V6T1Z4 Canada

## Abstract

Biofilms represent a multicellular growth state of bacteria that are intrinsically resistant to conventional antibiotics. It was recently shown that a synthetic immunomodulatory cationic peptide, 1018 (VRLIVAVRIWRR-NH_2_), exhibits broad-spectrum antibiofilm activity but the sequence determinants of antibiofilm peptides have not been systematically studied. In the present work, a peptide library consisting of 96 single amino acid substituted variants of 1018 was SPOT-synthesized on cellulose arrays and evaluated against methicillin resistant *Staphylococcus aureus* (MRSA) biofilms. This dataset was used to establish quantitative structure-activity relationship (QSAR) models relating the antibiofilm activity of these peptides to hundreds of molecular descriptors derived from their sequences. The developed 3D QSAR models then predicted the probability that a peptide would possess antibiofilm activity from a library of 100,000 virtual peptide sequences *in silico*. A subset of these variants were SPOT-synthesized and their activity assessed, revealing that the QSAR models resulted in ~85% prediction accuracy. Notably, peptide 3002 (ILVRWIRWRIQW-NH_2_) was identified that exhibited an 8-fold increased antibiofilm potency *in vitro* compared to 1018 and proved effective *in vivo*, significantly reducing abscess size in a chronic MRSA mouse infection model. This study demonstrates that QSAR modeling can successfully be used to identify antibiofilm specific peptides with therapeutic potential.

## Introduction

Biofilms represent a multicellular state of bacterial growth that can form on virtually any surface in which bacteria accumulate in structured aggregates encased by an extracellular matrix^1^. Biofilms are responsible for up to 65% of all infections and are associated with a variety of chronic infections in humans^2,3^. The bacteria within a biofilm are phenotypically distinct from planktonic bacteria and have substantially altered transcriptional profiles with one of the most notable distinctions being that bacteria within a biofilm are significantly (10- to 1000- fold) more resistant to conventional antibiotics and disinfectants^4^. To date no antibiofilm compounds have been approved for clinical use, highlighting the urgent need to develop novel anti-infectives that specifically target biofilms and enable treatment of biofilm-associated infections.

Our group recently identified short cationic peptides with antibiofilm activity^5–8^ that share many structural features with well-characterized antimicrobial peptides such as significant positive charge, large proportion of hydrophobic residues and amphipathicity^9,10^. Previous publications have employed various sequence optimization strategies aimed at enhancing the overall potency of antimicrobial peptides^11,12^. Most of these studies focused on small libraries of peptides in which modifications were made to remove residues that detracted from antibacterial activity (e.g. acidic and polar amino acids in the hydrophobic face) while maintaining basic and hydrophobic residues (especially Trp), known to contribute to overall potency. Unfortunately these strategies have shown limited success and rarely result in substantial improvements in antibacterial activity. Additionally, the activity of antibiofilm peptides is clearly independent of their antimicrobial activity against planktonic organisms^7^. Thus the fundamental structure and sequence requirements that govern the antibiofilm activity of peptides are poorly understood.

Here we exploited an unbiased quantitative structure-activity relationship (QSAR)-based methodology to systematically investigate the antibiofilm activity of peptides in terms of their chemical “descriptors” that serve as a surrogate for the three-dimensional structures of peptides^11,13–15^ and modeled the antibiofilm activities based on these descriptors. A molecular descriptor can be defined as the transformation of the chemical information within a given molecule (in this case a polypeptide sequence) into a set of numerical values based on the physico-chemical properties of the constituent amino acids and their relative positioning, which can then be used to model the activities of interest^16^. In this sense, the modeled structures take into account the exact composition of all atoms of the peptide in three-dimensional space. We previously showed that the QSAR modeling of antimicrobial peptides relating structure (as represented by descriptors) to activity was quite accurate in classifying peptides with antibacterial activity towards planktonic cells^11^. As a starting point, peptide 1018, also known as innate defence regulator (IDR)-1018 served as the template polypeptide sequence for our antibiofilm peptide screening strategy. This peptide is a 12-residue synthetic derivative of the natural bovine HDP bactenecin that displays a wide range of biological activities including pro and anti-inflammatory functions^17^ and potent broad spectrum antibiofilm activity^5^. Using a combination of SPOT-synthesized peptide arrays and high-throughput biofilm inhibition assays, we systematically quantified the antibiofilm activity of 96 peptides that were single amino acid substitution variants of 1018.

The optimal molecular descriptors of the single amino acid variants of 1018 were subsequently calculated, and used to model the experimentally determined antibiofilm activities. The best QSAR model was then employed to predict the most likely antibiofilm peptide sequences from a virtual peptide library consisting of 100,000 sequences *in silico*. A subset of the predicted sequences were synthesized and tested for their antibiofilm activity to confirm the accuracy of our QSAR predictions and resulted in the identification of several novel antibiofilm peptides including a considerably improved sequence with enhanced antibiofilm potency.

The reported methodology will assist further development of novel peptides with antibiofilm activity and enhanced therapeutic potential to address the growing concern of biofilm-associated infections.

## Results

### Antibiofilm screen of 1018-derived peptides

**Figure 1:**
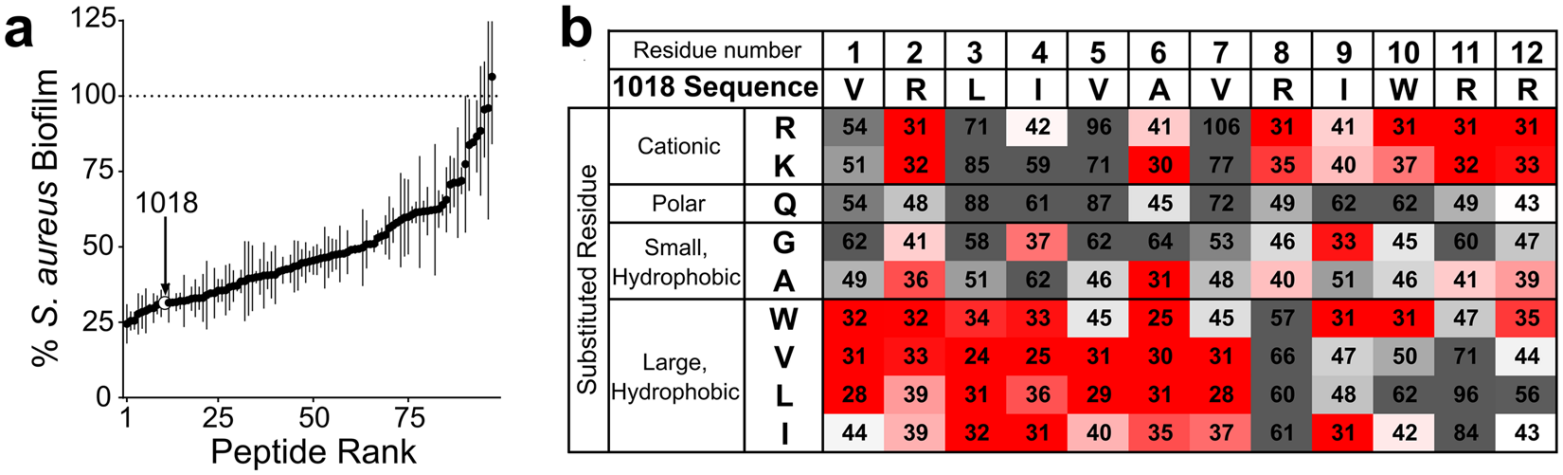
Distribution of antibiofilm activities of SPOT-synthesized 1018 single amino acid substitution peptides comprising the Training Set. The antibiofilm activities of the peptides in the Training Set were used for the initial QSAR models. The antibiofilm activity each peptide was evaluated against *S. aureus* (MRSA SAP0017) at a concentration of ~6.25 μM and the 1018 derivatives exhibited a large range of activities (**a**). The percentage of biofilm inhibited by SPOT-synthesized 1018 (31%) is indicated. When all the percentages of biofilm inhibition were plotted as an amino acid substitution matrix, those residues that contributed to the antibiofilm activity of 1018 became apparent (**b**). Each box represents an individual peptide shaded from the most active sequences (top 25^th^ percentile, in red) to moderately active (white) to least active (bottom 75^th^ percentile, in grey). The numbers in the boxes are the percent residual biofilm after treatment with peptide. See also Table S1.

Previously identified antibiofilm peptides by our group have been primarily composed of cationic and hydrophobic amino acids which are also present in high abundance in natural host defence peptides^9,10^. Notably, three of our most widely studied peptides 1018, 1002 and HH2 are comprised of only nine amino acids: Arg, Lys, Glu, Gly, Ala, Trp, Val, Leu and Ile^17,18^. Based on this knowledge, we chose to focus on these residues to generate the single amino acid substitution library of 1018 which concentrated our peptide design efforts towards amino acids found in known antibiofilm sequences. A 96 peptide, SPOT-synthesized, single amino-acid substitution library of 1018 (Supplementary Table S1) was tested for activity against MRSA (clinical isolate SAP0017) biofilms revealing a distribution of antibiofilm activities ranging from slightly more active than the parent peptide, 25% vs. 31% residual biofilm, to completely inactive (Fig. 1a). These activities were plotted as a substitution matrix which revealed those residues that were most important for antibiofilm activity and residues that could be mutated to enhance or maintain the antibiofilm potency and/or tune specific physico-chemical properties (Fig. 1b). For instance, hydrophobic residues appeared to be largely permitted in the seven N-terminal amino acid positions. In particular, substituting any of the hydrophobic residues at positions of Leu-3, Val-5 and Val-7 for anything other than a large hydrophobic residue substantially reduced the antibiofilm activity while Ile-4 could be substituted with large hydrophobic residues as well as Gly and Arg, although these derivatives were slightly less active. Notably, virtually all tested amino acids could be substituted for Arg-2 without sacrificing much of the antibiofilm potency while all substitutions at Ala-6 were well tolerated except for Gly. Conversely, at the C-terminus of 1018, substitutions for cationic residues were generally allowed, particularly for Arg-8 and Arg-11 where replacements with Lys appeared as quite favorable. The positions of Ile-9 and Trp-10 allowed slightly more diverse amino acid substitutions as replacements of Gly and Trp at position 9 resulted in retention of antibiofilm activity; similarly, placing cationic residues at position 10 appeared suitable.

Such structure-activity relationship information derived from the experimental substitution matrix could be used to conduct sequence-based design of 1018 derivatives by means of single and multiple amino acid substitutions, an approach recently applied to synthetic host defence peptides 1002 and HH2^18^. However, such conventional sequence-based approaches typically result in peptides with only modestly improved activity profiles and any optimized sequence would differ from the parent peptide by only a few residues. Here we sought to further our understanding of the underlying structure-activity relationships that define the antibiofilm properties of peptides and to discover improved peptide variants. Therefore we chose to use QSAR modeling for predicting antibiofilm activity of synthetic peptides based on their actual three dimensional structures, rather than primary sequences alone.

### Molecular descriptors and model training

Considering the large number (~2500) of molecular descriptors initially generated on the basis of 3D structures of the studied peptides, pruning techniques were applied to identify a reduced set of QSAR parameters to be used for final modeling. To avoid redundancy, only descriptors with less than 80% cross-correlation were considered. For those, forward stepwise analysis was performed to select variables with the most significant discrimination potential.

Furthermore, we applied several discrimination techniques, of which Linear Discriminant Analysis was selected to model the antibiofilm activity of peptides, similar as in our previous studies^19^. Various active/inactive cut-offs were also considered and the optimal value was defined at ~top 5 criteria, which was then used to model the Training Set (i.e. 5% of the most potent peptides were considered actives, and the remaining 95% were deemed as inactives for training purposes). For additional information, see Supplementary Note on Statistical analysis and data modeling.

We chose a clarification QSAR model that utilized only seven molecular descriptors (Table 1) to discriminate the Training Set into actives and inactives. The equation is shown below with all term multipliers (weights for the corresponding normalized descriptors in the final solution):

1 $$\begin{array}{ccc}{\boldsymbol{Class}} & = & 0.272+1.472\times {\rm{TDB02s}}+0.8745\times {\rm{RDF040v}}+0.4806\times {\rm{RDF130s}}\\  &  & +\,0.699\times {\rm{Mor09v}}+0.5295\times {\rm{Mor02s}}-0.495\times {\rm{Mor06s}}-0.542\times {\rm{HATS0s}}\end{array}$$

**Table 1:** Molecular Descriptors used in the QSAR models to define the antibiofilm activity of synthetic peptides.

Descriptor Name	Family	Description
TDB02s	3-Dimensional Autocorrelation^40^	3D Topological distance based descriptors - lag 2 weighted by Intrinsic-state.
RDF040v	Radial Distribution Function (RDF) Descriptor^41^	Chemical information extracted from geometrical information using a Radial Distribution Function - 040/weighted by van der Waals volume.
RDF130s	RDF Descriptor^41^	Radial Distribution Function - 130/weighted by Intrinsic-state.
Mor09v	3D-Molecule Representation of Structure based on Electron diffraction (3D-MoRSE) descriptor^42,43^	Similar to RDF descriptors but particularly sensitive to short distance atomic pairs. Signal 09/weighted by van der Waals volume.
Mor02s	3D-MoRSE descriptor^42,43^	Signal 02/weighted by Intrinsic-state.
Mor06s	3D-MoRSE descriptor^42,43^	Signal 06/weighted by Intrinsic-state.
HATS0s	Geometry, Topology, and Atom-Weights AssemblY (GETAWAY) descriptor^44,45^	Geometric descriptor that encodes positional information within a structure. Leverage-weighted autocorrelation of lag 0/weighted by I-state

A brief description of each descriptor is provided. For additional information, see references indicated.

This QSAR model proved to be statistically significant at a p value of <0.0001. Therefore, considering that the model was trained using the antibiofilm activity of a large data set of peptides with quite similar sequences, an accuracy of >90% obtained for the Training Set was considered as satisfactory. We also show in Table 2 most of the parameters usually considered for evaluating the performance of newly proposed QSAR models^20^. These results were deemed appropriate, as evidenced by the established false positive rate (fp_rate_) of ~11% for the training phase.

**Table 2:** Prediction performance for Linear Classification QSAR models.

Sets	N	MCC	Q_T_*	fp_rate_*	S_e_*	10 CV
Training Set	97	0.73	90.7	11.1	91.1	87.44
Experimental Validation Set	108	0.65	88.9	12.5	89.1	—

N – number of peptide sequences in the set. MCC – Matthews’ correlation coefficient to measure quality of binary classification. Q_T_* - Model accuracy percentage. fp_rate_* - False positive rate. S_e_* - Sensitivity ‘hit rate’. 10 CV – 10 fold cross validation.

### Model testing and *in silico* screen of the Virtual Peptide Set

To assess the predictive ability of the QSAR model, a 10 fold cross-validation was performed using 90% of all peptides for model training and the remaining 10% of peptides considered for validation. This was repeated ten times resulting in 10 separate refined QSAR models. The resulting averaged classification accuracy for the ten cross validation runs was established as 87.44% which demonstrated acceptable model validation and no significant over-training (Table 2). Once validated, the model was used to predict the antibiofilm activity of 100,000 virtual peptide sequences generated for the Virtual Set (see Supplementary Note on Virtual set sequence requirements). Those peptides were created using a customized Python script and their 3D structures were built and energy-minimized using a customized SVL script in the MOE package. Seven descriptors utilized by the linear function (Equation (1), Table 1) were then computed for the whole set of 100,000 virtual sequences and their potential to exhibit antibiofilm activity was ranked according to the QSAR model described above. After running the predictions, a set of 108 peptides (the Experimental Validation Set) was selected for SPOT-synthesis and experimental evaluation.

### Antibiofilm and antimicrobial activity of QSAR derived peptides

**Figure 2:**
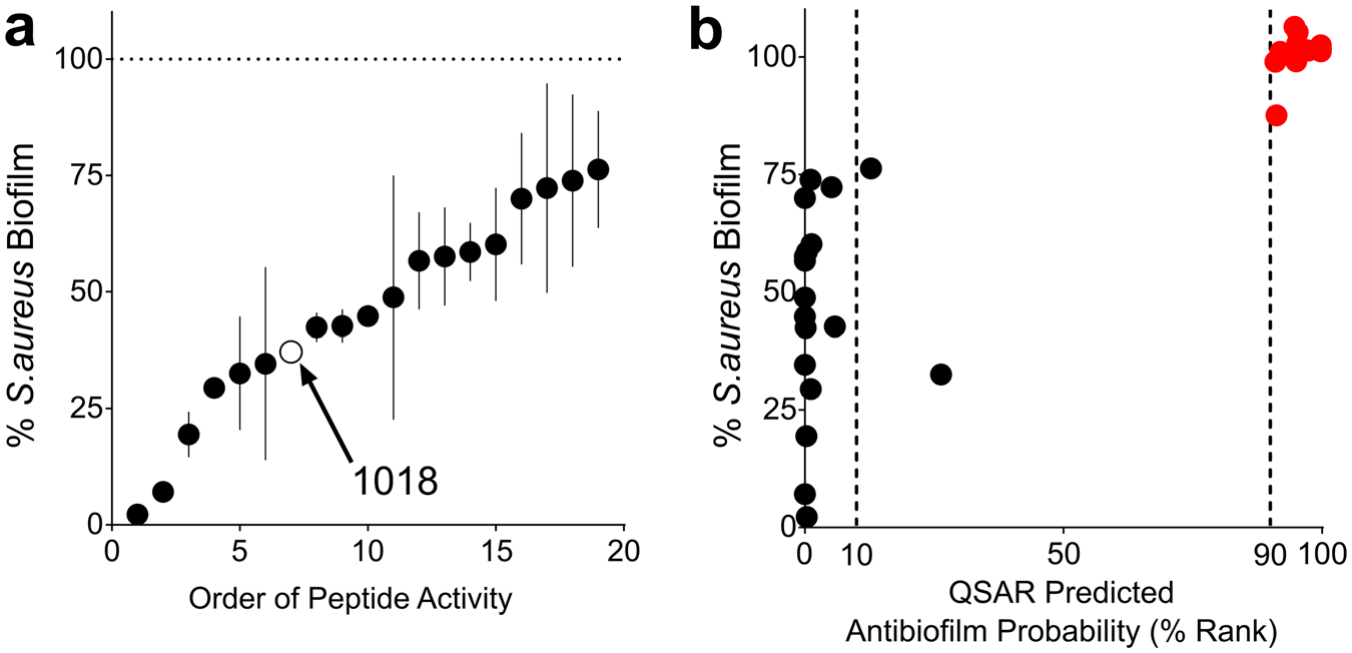
Antibiofilm activity of QSAR derived peptides towards MRSA biofilms. Of the 108 peptide sequences within the Experimental Validation Set that were SPOT-synthesized and evaluated for antibiofilm activity, 18 inhibited *S. aureus* (MRSA SAP0017) biofilm growth at the peptide concentration evaluated (**a**). At the same concentration, treatment with the other 90 peptides resulted in percent biofilm growth (+/−SEM) that overlapped with growth controls (not shown). A comparison of the measured antibiofilm activity of these 18 active peptides to the QSAR predicted antibiofilm probability reveals that most of these active sequences fall within the top 10th percentile of QSAR predicted sequences (**b**, black points). Additionally, all of the peptides from the bottom 10th percentile of QSAR predicted antibiofilm probability were inactive when evaluated for antibiofilm activity towards MRSA (**b**, red points). See also Table S2.

The Experimental Validation Set was comprised of 108 peptides that were randomly sampled from the 100,000 virtual sequences with both a high and low probability of exhibiting antibiofilm activity based on the classification by the QSAR model (Supplementary Table S2). These 108 sequences were SPOT-synthesized as before and were assessed for their antibiofilm activity using the same static microtitre plate assay used to screen the Training Set. At the highest peptide concentration evaluated, eighteen new peptides were identified that significantly inhibited MRSA biofilm growth compared to growth controls (Fig. 2a). Notably, almost all of these active peptides were within the top 10th percentile of probable antibiofilm sequences identified from the 100,000 sequences in the virtual set (Fig. 2b, black dots). In addition, all of the peptides from the experimental validation set that were predicted to have a low probability of exhibiting antibiofilm activity (90th percentile or lower) indeed did not inhibit MRSA biofilms under these conditions (Fig. 2b, red dots). Altogether, these results demonstrate that our modeling approach successfully identified novel antibiofilm peptide sequences and correctly classified inactive peptides under these experimental conditions.

**Figure 3:**
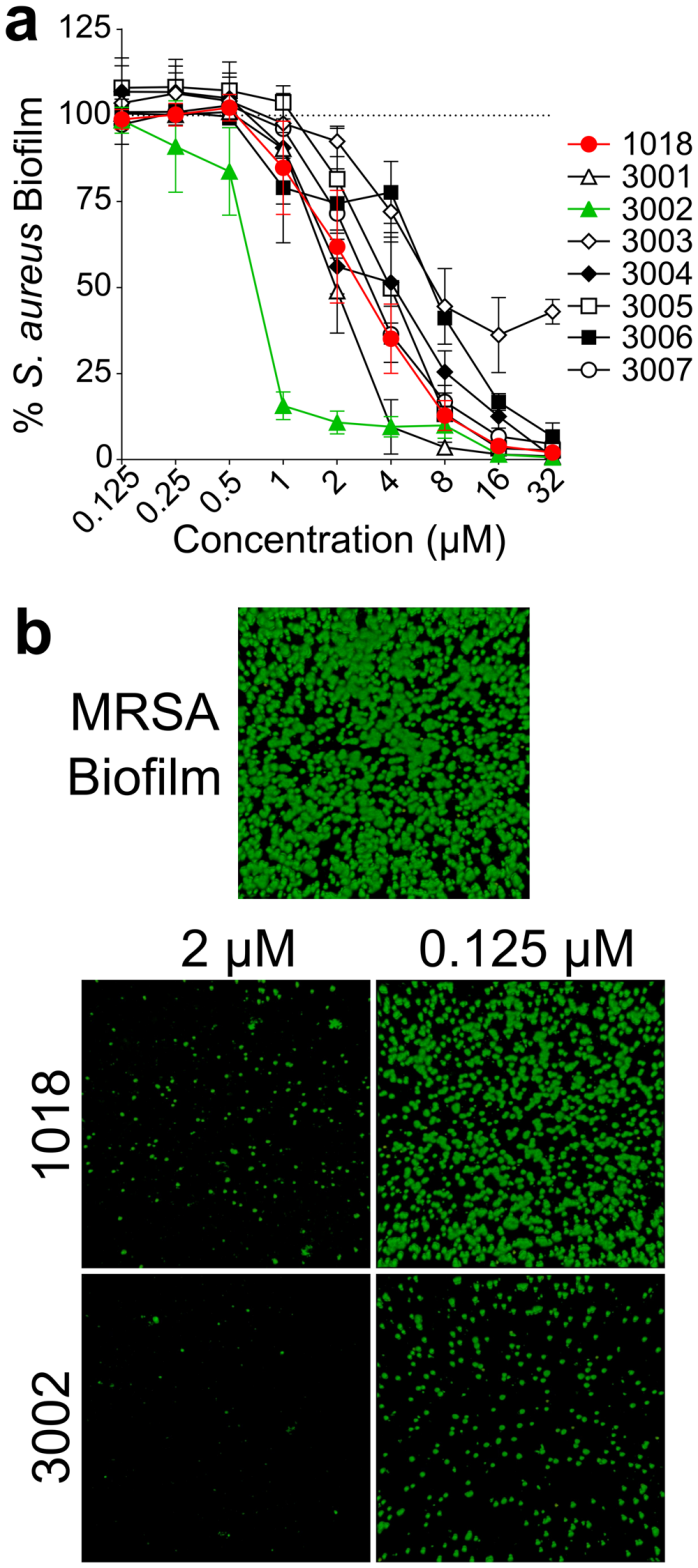
Antibiofilm activities of synthetic antibiofilm peptides identified by QSAR modeling. All peptides (3001–3007) were commercially synthesized to greater than 95% purity. The antibiofilm activity was initially evaluated in the static microtitre plate assay against *S. aureus* (MRSA SAP0017) and the residual biomass was stained with 0.1% crystal violet **(a)**. Most of the QSAR derived peptides demonstrated antibiofilm activity similar to the parent peptide, 1018 (highlighted in red), while one peptide, 3002 (highlighted in green), exhibited enhanced antibiofilm activity and substantially inhibited biofilm growth at peptide concentrations at low as 1 μM. MRSA biofilms were then grown in flow cells and treated with 1018 and 3002 to evaluate the ability of each peptide to eradicate pre-formed biofilms **(b)**. Peptide 3002 was found to substantially reduce preformed biofilms at 0.125 μM while 1018 was no longer effective at this same concentration.

The seven most active peptides identified from the screen of the SPOT-synthesized Experimental Validation Set (Fig. 2a) were synthesized to >95% purity and their antibiofilm activities were further characterized. The sequences and physico-chemical properties of these QSAR–predicted peptides (arbitrarily named 3001 to 3007) are presented in Table 3. When evaluated in the static microtitre plate assay, most of them exhibited antibiofilm activity similar to 1018 (Fig. 3a). Notably, peptide 3003 was not as potent as the other sequences with ~40% residual biofilm growth observed at a concentration of 32 μM. Conversely, peptide 3002 exhibited an 8-fold enhancement in antibiofilm potency compared to 1018, effectively inhibiting MRSA biofilm growth at concentrations as low as 1 μM (Fig. 3a).

**Table 3:** Peptide names, sequences and selected characteristics of the top antibiofilm peptides identified in this study.

Peptide	Sequence	Net Charge	Mwt (g/mol)	% Hydro-phobic	MIC (μM)	
					MHB	TSB with 1% Glucose
1018	VRLIVAVRIWRR-NH_2_	+5	1552.9	67	4	>64
3001	VIKWLLKILRAI-NH_2_	+4	1481.9	75	1	2
3002	ILVRWIRWRIQW-NH_2_	+4	1741.1	67	2	4
3003	WKKVQWLKRLLL-NH_2_	+5	1627.0	58	4	16
3004	IQRWWKVWLKVI-NH_2_	+4	1671.0	67	4	16
3005	RRQWRGWVRIWL-NH_2_	+5	1728.0	50	4	64
3006	IWLRLKVVLKRK-NH_2_	+6	1568.0	58	4	32
3007	VLKIKVKIWVVK-NH_2_	+5	1468.9	67	16	>64
Vancomycin	—	—	—	—	0.34	0.68

These seven peptides represent the most active antibiofilm sequences identified from the antibiofilm screen of the Experimental Validation Set of QSAR derived peptides. The MIC towards planktonic cells were determined in both Mueller Hinton Broth (MHB) and in Tryptic Soy Broth (TSB) supplemented with 1% glucose using the broth microdilution method. Reported MIC values (μM) are the mean value obtained from three individual biological replicates.

The antimicrobial activity of all the QSAR–predicted molecules was also assessed against planktonic (free swimming) MRSA in Mueller-Hinton broth (MHB), which is the medium typically used to determine MICs for synthetic peptides^21^ as well as tryptic soy broth (TSB) supplemented with 1% glucose, which is the same medium used in the antibiofilm screen. In MHB, most of the antibiofilm peptides yielded MIC values similar to that of 1018 (4 μM). When evaluated in TSB/1% glucose, all of the MIC values were at least 2–4 fold higher than the concentrations determined in MHB (Table 3). Notably, there was much greater variability in the MIC values as they ranged from 2 μM for 3001 to >64 μM for both 1018 and 3007. In particular peptide 3007 exhibited fairly specific antibiofilm activity. This is in agreement with previous observations^6,7^ that while there might be overlapping properties that govern antibacterial and antibiofilm potencies of synthetic peptides, these characteristics do not directly correlate.

To further evaluate the antibiofilm activity of the developed peptides in a complementary experiment, 2-day old MRSA biofilms grown in flow cell chambers were treated with peptides 3002 and 1018 and their ability to eradicate pre-formed biofilms was characterized. Biofilms grown in flow cells are generally considered to be a better model of biofilm formation and are more representative of naturally occurring biofilms^22^. Notably, both peptides effectively eradicated MRSA biofilms at a concentration of 2 µM (Fig. 3b). However, at a concentration of 0.125 μM, peptide 3002 effectively eradicated the 2-day old MRSA biofilms while biofilms treated with 1018 were virtually identical to untreated controls (Fig. 3b). Overall, our QSAR modeling approach successfully identified several novel antibiofilm peptides, whose primary amino acid sequences were distinct from our parent compound 1018.

### Cytotoxicity and immunomodulatory activity towards PBMCs

**Figure 4:**
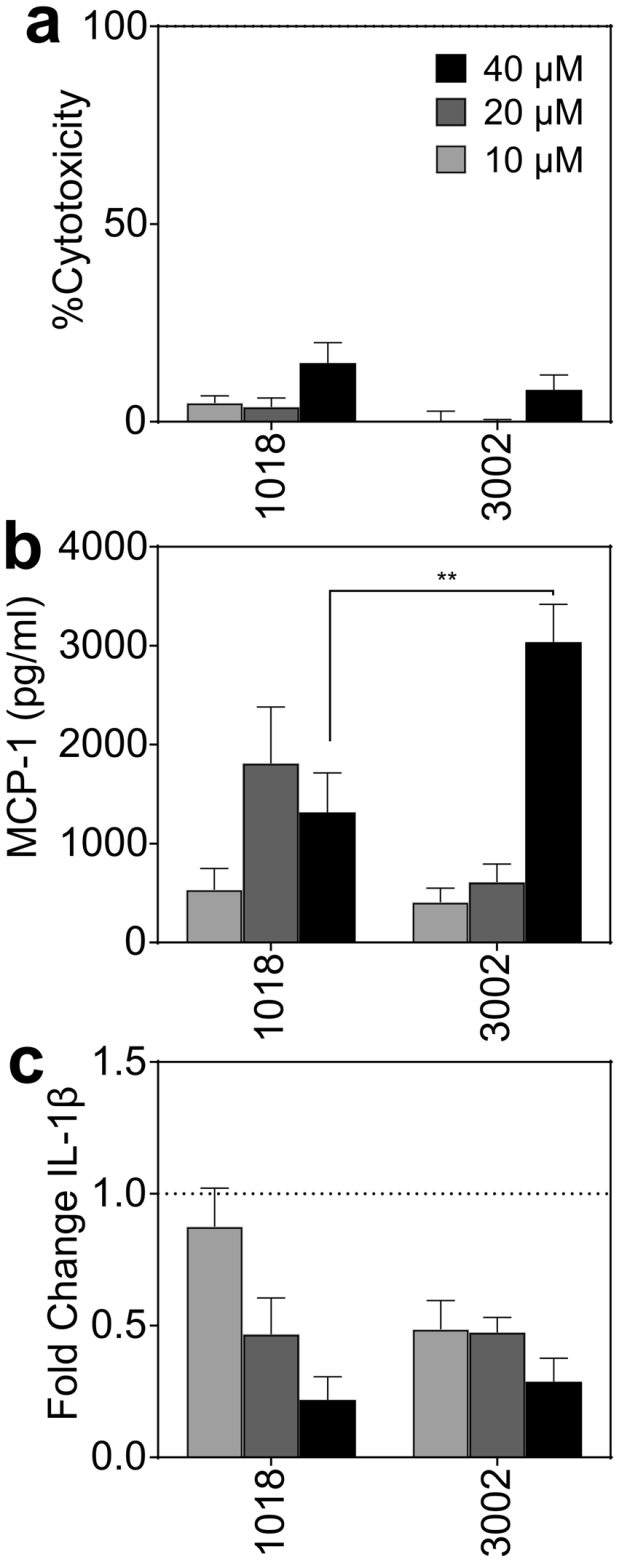
Toxicity and *in vitro* immunomodulatory activity of 1018 and 3002. Peptide cytotoxicity towards PBMCs was assessed by the LDH release assay **(a)**. Induction of MCP-1 chemokine production from PBMCs by peptide alone was evaluated **(b)** as well as the ability to suppress the production IL-1β from LPS-stimulated PBMCs **(c)**. The levels of both MCP-1 and IL-1β were determined by ELISA and shown are the average values from six biological replicates from individual donors. Statistical significance was calculated using a two-way ANOVA using a Bonferonni correction and comparing each concentration for each individual peptide (** p-value ≤ 0.01).

As peptide 1018 has been shown to possess immunomodulatory activity while being relatively non-toxic, we tested the capacity of antibiofilm compound 3002 to mediate such activity towards peripheral blood mononuclear cells (PBMCs). The cytotoxicity and immunomodulatory activity profile of 3002 was remarkably similar to that of 1018 (Fig. 4). Neither peptide caused substantial LDH release from PBMCs, causing less than 15% cytotoxicity at peptide concentrations as high as 40 µM (Fig. 4a). Remarkably, 3002 induced significantly more MCP-1 production at the highest peptide concentration evaluated (Fig. 4b) and suppressed proinflammatory interleukin (IL)-1β production from LPS-stimulated PBMCs in a concentration dependent manner to similar levels as seen for peptide 1018 (Fig. 4c).

### Efficacy of 3002 in a murine cutaneous abscess model

**Figure 5:**
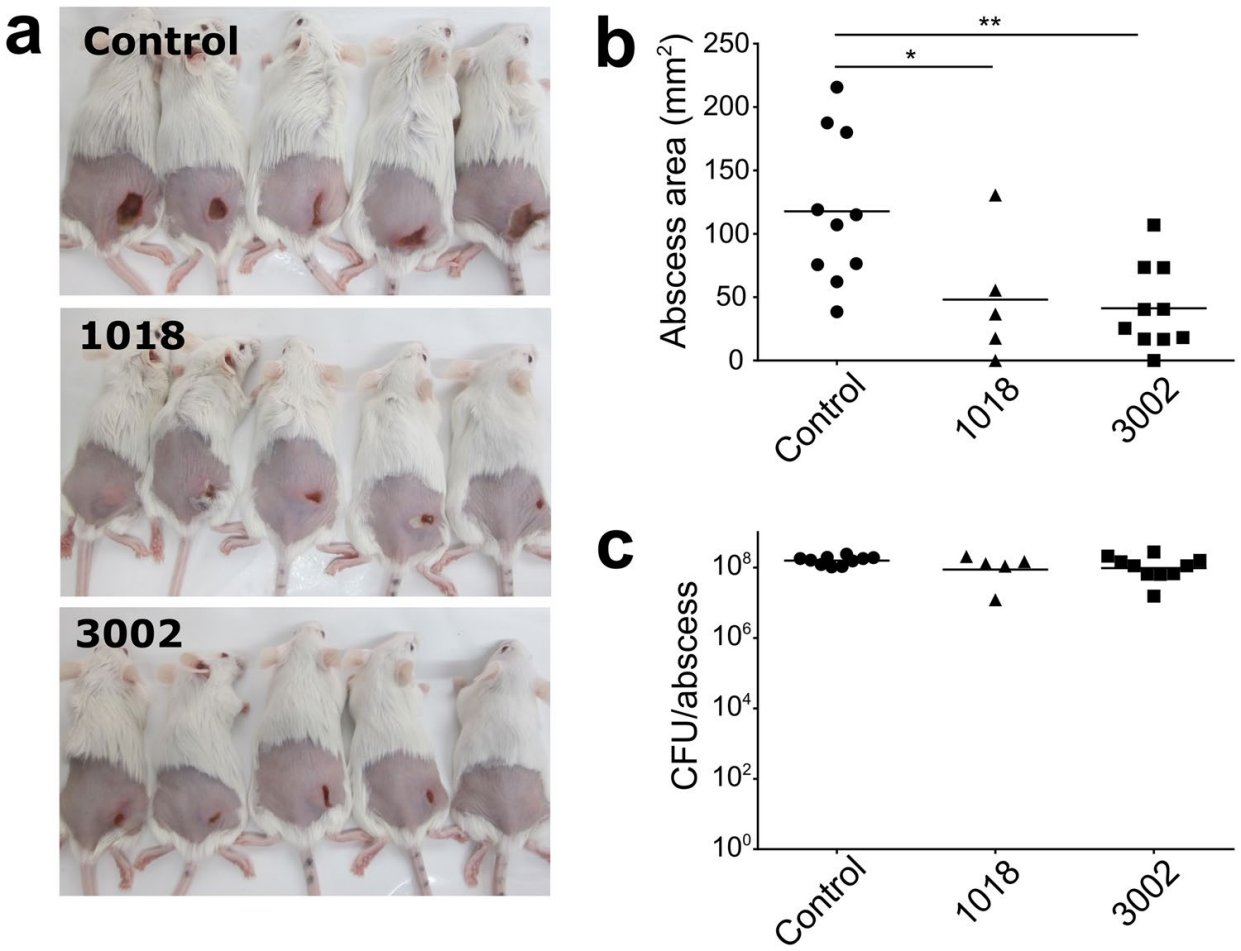
Effect of peptide treatment on abscess size and bacterial burden in an *in vivo* model of high density bacterial infection. CD-1 mice were injected with MRSA USA300 LAC at a density of 5 × 10^7^ CFU/50 µl to establish the abscess. After one hour, peptide (at 14 mg/kg) or vehicle (saline) control was injected intra-abscess and the abscess growth was monitored for 3 days. The representative photo of mice in the vehicle control group show prominent abscesses on the right flank while peptide treated abscesses were clearly smaller and less pronounced **(a)**. Quantification of the abscess sizes revealed that both 1018 and 3002 treatment significantly reduced the abscess size in peptide treated mice based on a one-way ANOVA analysis **(b)**. However, the bacterial burden within the peptide treated abscesses was unaffected by peptide treatment **(c)**.

To assess the efficacy of peptide 3002 against biofilm-like infections we further evaluated its activity *in vivo*. Only a few animal models are available that involve “biofilm” infections and they are often inconsistent, technically challenging and self-limiting, frequently resolving without antibiotic treatment^23^. Abscess infections, on the other hand, are populated by high bacterial densities and are recalcitrant to conventional therapies, features that are consistent with biofilm-associated infections. We have recently demonstrated that a mouse model of cutaneous abscess infections reproducibly and consistently forms abscesses with MRSA^24^ that are characterized by high bacterial densities and resistance to antibiotic treatment. Previously, we demonstrated that MRSA cutaneous abscess formation is regulated by the protective stringent response^24^, a conserved bacterial stress response elicited to survive harsh environmental conditions such as those encountered in the human host. This stress response involves the production of a secondary-messenger molecule, guanosine tetraphosphate (ppGpp), which is also required for biofilm initiation and maintenance^5^. Consequently, such infections are susceptible to the effects of synthetic antibiofilm peptides which have been shown to target ppGpp in biofilms^5,24^. Therefore, using this model as a proxy for biofilm-like infections, we infected mice subcutaneously with community associated MRSA strain USA300 LAC and administered 3002 or 1018 to assess the effect of peptide on abscess formation. In this model, administration of 14 mg/kg of either 3002 or 1018 via a single intra-abscess injection significantly reduced the lesion size by 2.8-fold and 2.4-fold respectively, compared to untreated controls (Fig. 5). While 3002 and 1018 demonstrated antibiofilm potency *in vitro*, their direct antimicrobial activity was different and media-specific (Table 3). Furthermore, as seen with the human peptide LL-37, direct antimicrobial activity *in vivo* is often antagonized by host serum proteins and divalent cations^25^. It is therefore notable that neither peptide had any effect on the bacterial load recovered from the abscesses (Fig. 5c), suggesting that the decrease in abscess size may be due to antibiofilm activity combined with additional immunomodulatory effects^17^ induced by the peptides (Fig. 4).

## Discussion

The increasing prevalence of antibiotic resistance in pathogenic bacteria^26^ coupled with the fact that many clinically relevant infections are caused by intrinsically resistant bacterial biofilms^27^ highlights the urgent need for the development of specific antibiofilm agents. A number of cationic peptides have recently been identified with broad-spectrum antibiofilm potency^5,8^, providing a basis for a potential therapeutic solution. The present study utilized a cheminformatics approach to establish structure-activity models based on measured antibiofilm activities of a peptide library derived from a previously reported antibiofilm peptide, 1018. This QSAR approach successfully generated accurate models that correctly classified the investigated peptides as either active or inactive. These QSAR solutions were then used to screen thousands of hypothetical peptide sequences *in silico* and resulted in the development of a number of novel antibiofilm peptides that substantially differed in their primary amino acid sequences yet exhibited enhanced activity against MRSA biofilms *in vitro* and proved effective *in vivo*. Overall, these results clearly demonstrate the usefulness of this approach to generate novel antibiofilm peptides with superior potencies.

It is important to note that the antibiofilm peptides identified in this study have all been optimized against MRSA biofilms. Even though the clinical relevance of MRSA biofilms is known^28,29^, developing an antibiotic with a broad-spectrum antibiofilm potency would be desirable. Importantly, peptide 1018 already demonstrated a broad spectrum antibiofilm activity against a variety of Gram- positive and negative bacteria^5^. Similarly, we are currently testing the identified peptide 3002 against a range of bacterial biofilms.

It has also been recently demonstrated that antibiofilm peptides can synergize with conventional antibiotics. For example, when peptide 1018 was used in combination with various antimicrobial agents, it decreased the amount of drug required to treat biofilms formed by *P. aeruginosa*, *Escherichia coli* and MRSA (among others) by up to 64-fold^30^. In another study, peptide DJK-6 demonstrated a potentiating effect on β-lactam antibiotics against multi-drug resistant carbapenemase-producing *Klebsiella pneumoniae*^31^. It is likely that such synergistic effects would be conserved with the latest peptide 3002, which should further decrease the amount of antibiotic required to treat biofilms formed by pathogenic bacteria.

It is important to emphasize that our modeling strategy only classified peptides as “active” or “inactive” and it did not take into account antibiofilm potency. Using our experimental approach, we were limited by the total amount of peptide that we could obtain from the SPOT-synthesized arrays and thus the highest peptide concentration that we evaluated in the microtitre assay was dictated by peptide stock solutions obtained from the arrays. We would expect that if we could assess the antibiofilm activity at higher peptide concentrations that more of the probable antibiofilm peptides would inhibit MRSA biofilm growth while those with low predicted probabilities would remain inactive. We also anticipate that it should be possible to iteratively improve the accuracy of QSAR models for antibiofilm peptides as more active sequences are reported in the literature and SPOT-synthetic methodology continues to generate relevant data. Furthermore, a database of biofilm-active antimicrobial peptides was recently established^32^ and it may be possible to incorporate these additional sequences into broadened QSAR solutions with improved predictive power.

Finally, it should be highlighted that many antibiofilm peptides possess immunomodulatory properties^18,33^ which makes them even more attractive treatment options for biofilm-associated infections. We would expect that an ideal peptide drug would target bacteria within the biofilm responsible for the infection while also enhancing the recruitment of important effector cells and suppressing the harmful effects of inflammation. Indeed, the QSAR identified antibiofilm peptide 3002 exhibited an immunomodulatory profile towards PBMCs similar to that of 1018, suppressing the LPS-induced production of a pro-inflammatory cytokine and exhibiting enhanced capacity for chemokine induction by peptide alone. While these two examples would suggest that there is overlap among peptides with antibiofilm and immunomodulatory properties, it is currently unknown whether the sequence requirements underlying these two activity types are the same and QSAR may also be employed to further explore the corresponding structure-activity relationship biases.

Ultimately, the results of the current work clearly demonstrate the potential of using QSAR methodology for identifying synthetic antibiofilm peptides that bring a promise of combating life-threatening bacterial infections in a variety of ways, including direct microbial killing, immune-modulation and critical disruption of biofilms.

## Methods

### Peptide synthesis and stock solution preparation

SPOT-synthesized peptides were prepared by Kinexus Inc. (Vancouver, BC. Canada) and were obtained as free molecules that had been released from the cellulose membrane by ammonia gas treatment^34^. A library of experimental peptides derived from the parental sequence 1018 was used consisting of 96 single amino acids substitution variants (Supplementary Table S1), where each residue in 1018 was systematically replaced with nine amino acids (R, K, Q, G, A,W, V, L and I) that have previously been associated with antibiofilm potential in synthetic peptides. Each peptide spot (~100 nmol density, 60–70% purity) was dissolved in 200 μl of sterile water to generate a stock peptide solution. The concentration of a few SPOT-peptide stock solutions was determined by measuring the Trp absorbance at 280 nm on a NanoDrop instrument and calculated by Beer’s law using a Trp extinction coefficient of 5500 M^−1^ cm^−1^. This concentration (~250 µM) was then assumed to be representative of all the SPOT-peptide stock solutions. Synthetic 1018 at >95% purity was obtained from CPC Scientific (Sunnyvale, CA) while all other synthetic peptides (>95% purity) were obtained from Genscript (Piscataway, NJ). The C-terminal carboxyl groups of all >95% pure peptides were amidated to remove the negative charge. Stock solutions of >95% pure peptide were prepared to 2 mg/ml in sterile water then diluted to the appropriate working concentration on the day of the experiment.

### Minimal inhibitory concentration (MIC) determination

The MIC of the synthesized peptides was determined in both Mueller Hinton Broth (MHB) and Tryptic Soy Broth (TSB) supplemented with 1% glucose, using the broth microdilution method^21^ with slight modifications. Briefly, 10 μl of a 10× concentrated peptide stock solution, vancomycin antibiotic, or vehicle control (water) was placed in the bottom of a well in a 96-well polypropylene round bottom plate (Corning Inc. Corning, NY). A bacterial suspension of *S. aureus* MRSA (SAP0017, clinical isolate kindly provided by Dr. Tony Chow, Vancouver General Hospital) was prepared from an overnight culture to a final cell density of ~5 × 10^5^ CFU/ml and 90 μl of this was added to each well. The plates were incubated at 37 °C and the following day the MIC was visually determined as the lowest concentration of peptide that resulted in no visible growth in the well.

### Antibiofilm activity screen

The SPOT-synthesized peptides were evaluated for their antibiofilm activity *in vitro* as described previously^18^. Briefly, 5 μl of SPOT-peptide stock solution or a corresponding serial dilution was placed in a 96-well polypropylene round bottom plate (Corning Inc. Corning, NY) and 95 μl of a 1/100 dilution of an overnight culture of MRSA SAP0017 in TSB supplemented with 1% glucose was added to each well. The plates were incubated overnight at 37 °C to allow for bacterial growth and biofilm maturation. After 24 hours, the media containing planktonic bacteria was rinsed from each well with distilled water and the remaining biomass was stained with 0.1% crystal violet. The unbound dye was rinsed away and the biofilm bound dye was suspended in 70% ethanol and the absorbance at 595 nm was recorded on a plate reader. Percent biofilm inhibition was calculated by comparison to the amount of crystal violet stain in the untreated controls (defined as 100%) and the sterility control wells (defined as 0%). Results from three separate biological replicates were averaged and outliers were identified using the modified Thompson Tau test and removed from the analysis. The antibiofilm activity of all SPOT-derived peptides was assessed at multiple concentrations (up to five 2-fold dilutions from the stock solution) and the peptide concentration yielding the largest difference in percent inhibition of biofilm growth was used in subsequent analysis. The top seven QSAR derived peptides identified in the Experimental Validation Set with the most potent antibiofilm activity were synthesized to 95% purity and their antibiofilm activity was assessed in the same manner.

### Experimental data processing and peptide set definitions

The set of 96 single amino acid substituted peptides derived from a parental compound 1018 as well as 1018 itself (Supplementary Table S1), was prepared for modeling purposes (described below) and used as a Training Set for initial modeling. The experimental values were defined as the percent of biofilm inhibition which were determined as described above. A *de novo* set of 100,000 virtual peptides (referred to as the Virtual Set) was generated using defined sequence constraints that ensured that the Virtual Set sequences would have similar physico-chemical characteristics to those of the parent peptide 1018 (Supplementary Table S3). The Virtual Set sequences were generated using custom script within the Python environment and further optimized using custom SVL scripts for the MOE software (Molecular Operating Environment 2013.08. Chemical Computing Group Inc. Montreal, Canada). Peptides conforming to this set were used as the Test Set to evaluate *in silico* system’s ability to predict new sequences. Following computer ranking of the top antibiofilm peptides in the Virtual Set, a sampling of 108 sequences, including 1018 itself (termed the Experimental Validation Set) with varying predicted potencies against biofilms were SPOT-synthesized and their antibiofilm activity was experimentally determined.

### Molecular descriptors computation

The Training Set peptide sequences were stored in a SDF file using MOE. The peptide structures were then energy-minimized to establish their most probable 3D conformations using standard MOE built-in capabilities and a custom SVL script. Molecular descriptors for the Training Set peptides were calculated using MOE and Dragon 6.0 software (TALETE srl. 2011. Milano, Italy). Additionally, inductive QSAR molecular descriptors were computed using a custom SVL script^35–37^. All these molecular parameters have been previously used for chemoinformatic modeling of antimicrobial potentials of short cationic peptides^13^ and in other areas^38^. In total, more than 2500 molecular descriptors were calculated for each studied peptide. The molecular descriptors were filtered to exclude those with zero variance and low occurrence (represented in less than 25% of compounds). Also, non-discriminatory and highly correlated molecular descriptors were eliminated. The remaining QSAR parameters were investigated for their ability to correctly assign Training Set peptides into ‘active’ or ‘inactive’ categories based on a defined threshold value (See Supplementary Note on Statistical Analysis and Data Modeling). As the result, seven of the most relevant molecular descriptors were selected and then used to train QSAR models for the Training Set and to predict antibiofilm potential of molecules in the external Virtual Set.

### *In silico* testing and *in vitro* screening of novel peptides

To test the predictive ability of the models, all peptides in the Virtual Set were predicted 10 times (using 10 cross validated training models) and the resulting predictions were summed and ranked together in a single list according to their probability of being active or inactive. A set of peptides from within the 100,000 in the Virtual Set were selected and SPOT-synthesized on cellulose arrays to evaluate the ability of the trained binary QSAR models to distinguish active from inactive peptide variants. This Experimental Validation Set consisted of 108 peptides distributed throughout the expected activities in Virtual Set including 54 peptides from the top 10% of predicted antibiofilm molecules and 14 peptides from the bottom 10% of the ranked list (Supplementary Table S2). Finally, the antibiofilm activity of the compiled Experimental Validation Set was evaluated against MRSA using the crystal violet assay (described above).

### Antibiofilm activity in flow cells

The most active peptide (molecule 3002) identified in the Experimental Validation Set was further tested against MRSA SAP0017 biofilms grown in flows cells as described previously^5,18^ and compared to the antibiofilm activity of the parent peptide 1018. Bacterial biofilms were grown in BM2 media (62 mM potassium phosphate buffer, pH 7, 7 mM (NH_4_)_2_SO_4_, 2 mM MgSO_4_, 10 μM FeSO_4_ and 0.4% (wt/vol) glucose) for 48 hours followed by treatment with peptide (2 or 0.125 µM) for an additional 24 hours. The samples were stained with SYTO-9 and propidium iodide fluorescent dyes to image total and dead cells, respectively. Flow-cell images were acquired on an Olympus FluoView FV1000 confocal laser scanning microscope (Olympus Corp. Tokyo, Japan) and were analyzed with the Fiji software package^39^.

### Toxicity and *in vitro* immunomodulatory activity

The cytotoxicity and immunomodulatory activity of both 1018 and 3002 was assessed as described previously^18^. Briefly, peripheral blood mononuclear cells (PBMCs) were collected from healthy volunteers in accordance with the University of British Columbia ethics guidelines. PBMCs were plated in Costar 96-well flat bottom tissue culture treated plates at a final density of 1 × 10^6^ cells/ml in RPMI media supplemented with 10% fetal bovine serum. Both peptides were tested at final concentrations of 40, 20 and 10 µM. Cytotoxicity was assessed using the Cytotoxicity Detection Kit (Roche Diagnostics, Basel, Switzerland) which measures lactate dehydrogenase activity released from damaged cells. Immune modulation was characterized by measuring the production of MCP-1 chemokine by induced by peptide alone as well as the suppression of interleukin (IL)-1β from PBMCs stimulated with 10 ng/ml *Pseudomonas aeruginosa* PAO1 lipopolysaccharide. Levels of MCP-1 and IL-1β were quantified by enzyme linked immunosorbent assays (ELISAs) using kits from eBioscience Inc. (San Diego, CA).

### MRSA *in vivo* murine cutaneous abscess model

The *in vivo* activity of the identified antibiofilm peptide 3002 and the parental compound 1018 was evaluated using a recently described murine abscess model of chronic MRSA infections^24^. All animal experiments were performed in accordance with The Canadian Council on Animal Care (CCAC) guidelines and were approved by the University of British Columbia Animal Care Committee. Briefly, the fur on the backs of 6-week old female CD-1 mice (Charles River Laboratories, Wilmington, MA) was removed by shaving and subsequent treatment with depilatory cream. MRSA USA300 LAC was grown to an optical density at 600 nm of 1 in tryptic soy broth (TSB) at which point cells were washed twice with sterile PBS and then suspended in PBS to a final cell density of 5 × 10^7^ CFU/50 μL. Mice were infected with 50 μL of bacteria subcutaneously on the right flank of the back and 1 hour later, given saline (for controls) or treated with 14 mg/kg peptide (1018 or 3002) via intra-abscess injection. Mice were monitored daily and their weight as well as abscess lesion sizes (measured using a caliper) were recorded every 24 hrs. Visible dermonecrosis or white lesions (filled with pus) were considered as part of the abscess lesion while swelling/inflammation was disregarded in the abscess size measurements. Three days post-infection, abscesses were excised and homogenized using a Bead Ruptor 4 tissue homogenizer (OMNI International, Kennesaw GA) for 5 minutes and serially plated for viable CFU quantification.

### Data Availability

The datasets generated during and/or analysed during the current study are available from the corresponding author on reasonable request.

## Electronic supplementary material

Supplementary information
