# Supplementary material for: Computer-aided Discovery of Peptides that Specifically Attack Bacterial Biofilms
Source: Sci Rep. 2018 Jan 30;8:1871. doi: 10.1038/s41598-018-19669-4 (PMC5789975; doi:10.1038/s41598-018-19669-4)
Supplement: Supplementary file 1 — Supplementary information [file 41598_2018_19669_MOESM1_ESM.pdf]

Supplementary Information for "Computer-aided Discovery of Peptides that Specifically Attack Bacterial Biofilms"

Evan F. Haney[a], Yoan Brito-Sánchez[b], Michael J. Trimble[a], Sarah C. Mansour[a], Artem Cherkasov[b], Robert E.W. Hancock[a*]

[a]Centre for Microbial Diseases and Immunity Research, and [b]Vancouver Prostate Centre, University of British Columbia, Vancouver, British Columbia, V6T1Z4, Canada.

* Corresponding author – email – bob@hancocklab.com

Supplementary Table S1 – Sequences of single amino acid substitution variants of 1018 comprising the Training Set that were SPOT-synthesized and evaluated for their antibiofilm activity against *S. aureus*.

No.	Name	Sequence	No.	Name	Sequence	No.	Name	Sequence
1	1018	VRLIVAVRIWRR	34	1018-K2	VKLIVAVRIWRR	67	1018-V3	VRVIVAVRIWRR
2	1018-G1	GRLIVAVRIWRR	35	1018-K3	VRKIVAVRIWRR	68	1018-V4	VRLVVAVRIWRR
3	1018-G2	VGLIVAVRIWRR	36	1018-K4	VRLKVAVRIWRR	69	1018-V6	VRLIVVVRIWRR
4	1018-G3	VRGIVAVRIWRR	37	1018-K5	VRLIKAVRIWRR	70	1018-V8	VRLIVAVVIWRR
5	1018-G4	VRLGVAVRIWRR	38	1018-K6	VRLIVKVRIWRR	71	1018-V9	VRLIVAVRVWRR
6	1018-G5	VRLIGAVRIWRR	39	1018-K7	VRLIVAKRIWRR	72	1018-V10	VRLIVAVRIVRR
7	1018-G6	VRLIVGVRIWRR	40	1018-K8	VRLIVAVKIWRR	73	1018-V11	VRLIVAVRIWVR
8	1018-G7	VRLIVAGRIWRR	41	1018-K9	VRLIVAVRKWRR	74	1018-V12	VRLIVAVRIWRV
9	1018-G8	VRLIVAVGIWRR	42	1018-K10	VRLIVAVRIKRR	75	1018-W1	WRLIVAVRIWRR
10	1018-G9	VRLIVAVRGWRR	43	1018-K11	VRLIVAVRIWKR	76	1018-W2	VWLIVAVRIWRR
11	1018-G10	VRLIVAVRIGRR	44	1018-K12	VRLIVAVRIWRK	77	1018-W3	VRWIVAVRIWRR
12	1018-G11	VRLIVAVRIWGR	45	1018-L1	LRLIVAVRIWRR	78	1018-W4	VRLWVAVRIWRR
13	1018-G12	VRLIVAVRIWRG	46	1018-L2	VLLIVAVRIWRR	79	1018-W5	VRLIWAVRIWRR
14	1018-A1	ARLIVAVRIWRR	47	1018-L4	VRLLVAVRIWRR	80	1018-W6	VRLIVWVRIWRR
15	1018-A2	VALIVAVRIWRR	48	1018-L5	VRLILAVRIWRR	81	1018-W7	VRLIVAWRIWRR
16	1018-A3	VRAIVAVRIWRR	49	1018-L6	VRLIVLVRIWRR	82	1018-W8	VRLIVAVWIWRR
17	1018-A4	VRLAVAVRIWRR	50	1018-L7	VRLIVALRIWRR	83	1018-W9	VRLIVAVRWWRR
18	1018-A5	VRLIAAVRIWRR	51	1018-L8	VRLIVAVLIWRR	84	1018-W11	VRLIVAVRIWWR
19	1018-A7	VRLIVAARIWRR	52	1018-L9	VRLIVAVRLWRR	85	1018-W12	VRLIVAVRIWRW
20	1018-A8	VRLIVAVAIWRR	53	1018-L10	VRLIVAVRILRR	86	1018-Q1	QRLIVAVRIWRR
21	1018-A9	VRLIVAVRAWRR	54	1018-L11	VRLIVAVRIWLR	87	1018-Q2	VQLIVAVRIWRR
22	1018-A10	VRLIVAVRIARR	55	1018-L12	VRLIVAVRIWRL	88	1018-Q3	VRQIVAVRIWRR
23	1018-A11	VRLIVAVRIWAR	56	1018-I1	IRLIVAVRIWRR	89	1018-Q4	VRLQVAVRIWRR
24	1018-A12	VRLIVAVRIWRA	57	1018-I2	VILIVAVRIWRR	90	1018-Q5	VRLIQAVRIWRR
25	1018-R1	RRLIVAVRIWRR	58	1018-I3	VRIIVAVRIWRR	91	1018-Q6	VRLIVQVRIWRR
26	1018-R3	VRRIVAVRIWRR	59	1018-I5	VRLIIAVRIWRR	92	1018-Q7	VRLIVAQRIWRR
27	1018-R4	VRLRVAVRIWRR	60	1018-I6	VRLIVIVRIWRR	93	1018-Q8	VRLIVAVQIWRR
28	1018-R5	VRLIRAVRIWRR	61	1018-I7	VRLIVAIRIWRR	94	1018-Q9	VRLIVAVRQWRR
29	1018-R6	VRLIVRVRIWRR	62	1018-I8	VRLIVAVIIWRR	95	1018-Q10	VRLIVAVRIQRR
30	1018-R7	VRLIVARRIWRR	63	1018-I10	VRLIVAVRIIRR	96	1018-Q11	VRLIVAVRIWQR
31	1018-R9	VRLIVAVRRWRR	64	1018-I11	VRLIVAVRIWIR	97	1018-Q12	VRLIVAVRIWRQ
32	1018-R10	VRLIVAVRIRRR	65	1018-I12	VRLIVAVRIWRI			
33	1018-K1	KRLIVAVRIWRR	66	1018-V2	VVLIVAVRIWRR			

Supplementary Table S2 – Peptide sequences of the Experimental Validation Set and their QSAR predicted antibiofilm probability (% rank) from within the 100,000 total peptides in the Virtual Set. Peptide sequences that exhibited antibiofilm activity from the SPOT-synthesized array include (from the most active to the least active): 25, 8, 23, 38, 58, 12, 22, 48, 17, 11, 6, 9, 26, 41, 10, 46, 37 and 56 (highlighted in bold). These peptide activities are shown in Fig 2A in the main text. All other peptides did not inhibit MRSA biofilms at the peptide concentration evaluated compared to untreated controls.

Num.	Sequence	% Rank	Num.	Sequence	% Rank	Num.	Sequence	% Rank
1018	VRLIVAVRIWRR	-	36	KVIKIVLVRVVK	1.1	72	QQLRWKRVAKAI	53.4
1	QRWKKWKVLKLR	0.001	**37**	**IKWVLRKIVQII**	**1.1**	73	KKAIKVVAIGRI	55.7
2	KVVWWKVIIKVL	0.001	**38**	**IQRWWKVWLKVI**	**1.2**	74	GRVLKIVWRKGR	56.3
3	KIWLLKLRQRQK	0.003	39	VKWKGKVIVVQL	1.2	75	VVGLRVRWVRLW	59.3
4	WRIKKQWIQIIV	0.003	40	LKLKAILKIIRV	1.2	76	WAVRALKVKWAL	59.9
5	VARWKIIIAKLW	0.003	**41**	**LIVIQLLKKWWK**	**1.3**	77	LKILIAQAKKGL	60.7
**6**	**VQWIQIVVWRKR**	**0.003**	42	RVKAIKWRKIVV	3.3	78	VWLAQKIGKWIW	66.1
7	KVQIIKQLIAKK	0.007	43	IKIIWKALGQVI	3.6	79	AVAKWALKLWKQ	66.8
**8**	**ILVRWIRWRIQW**	**0.007**	44	GKLKIKVKLGIA	4.3	80	RGRLKQKWWRRL	67.4
**9**	**VIKVLIKRWLKL**	**0.009**	45	KGKIRKIVLIRR	4.6	81	VKGAIKRGIWVK	70.2
**10**	**RRIIKILLWKLR**	**0.01**	**46**	**WIIRWIKIWLKI**	**5.2**	82	VIRAKAVWGWVK	70.9
**11**	**KKWQLLIKWKLR**	**0.01**	47	IVKKVKLIWGVK	5.3	83	KIWGLLKLGIAL	73.1
**12**	**IWLRLKVVLKRK**	**0.012**	**48**	**IQLKLIWVKRKW**	**5.8**	84	LAGLIVKWAGVR	74.1
13	IILKRVQVQKIK	0.012	49	VAKVKKARWRLR	6.4	85	AVKWLGWILAKK	74.9
14	KRIKKLLKVVLK	0.014	50	RQVRVKRWRARW	7.0	86	VARAVQKRWRKK	75.5
15	QQKVIRLLWKAK	0.015	51	KIVQKKLRLVVI	7.1	87	IVKWIAQWKLVG	80.2
16	KRLQWVKVKKIR	0.016	52	QIIKVVWRAVII	7.4	88	VKAKRWKWAQLA	81.0
**17**	**VLQIKKVLRLLL**	**0.017**	53	QVVVKKKAIQVV	7.6	89	LLIAGKWWKLAI	82.7
18	RIWRRAWKARWK	0.018	54	IRILVLRKAIIV	9.3	90	QKIGRAVIWKVK	83.4
19	KIVIRIILQVIK	0.019	55	KKQKKIWRRILV	11.1	91	RAIIKQRWQRRW	84.6
20	KIKLIQKQLRIK	0.02	**56**	**LWQLWLKLKLKG**	**12.8**	92	WVGVIIKWGLKL	85.6
21	WWIKIVVIRVRR	0.1	57	LQRVIWQKWRKV	22.0	93	KKIRQWGKAAAW	88.1
**22**	**VLKIKVKIWVVK**	**0.2**	**58**	**RRQWRGWVRIWL**	**26.3**	94	RLIQWGWKIWAV	91.0
**23**	**WKKVQWLKRLLL**	**0.3**	59	RGARVIRWKLRR	27.9	95	QLRVAWKRAWWA	91.2
24	IKIVRRAKIIIW	0.3	60	IAWQLLWGWRVR	31.0	96	RARIGIWKKWWA	91.9
**25**	**VIKWLLKILRAI**	**0.4**	61	KRKQWKLWVRQI	32.2	97	IQIQLVKRWAVI	92.2
**26**	**GLIIKIIKKRLW**	**0.4**	62	KLLGILKQAIVV	35.8	98	KAVKKGRRAIVV	94.7
27	IQIWIIRVIWRW	0.4	63	WQGWAKIWVVRI	36.3	99	VLLRVGARIVVG	95.0
28	LLKLKQKGIVIA	0.4	64	LKKIIVQAVGLI	38.6	100	GAKIIRKVAQVA	95.2
29	IIKWIVVRQIRK	0.5	65	IGQVVLVKIKIA	40.2	101	RLAKRKGQAIWV	95.3
30	WLKRIVKVVVLK	0.5	66	ALAIKVWIKILQ	45.3	102	IKAAKAGQWRRV	97.3
31	KVIQWIIVRRVL	0.5	67	VIAKIVLLRAGL	45.4	103	ALLAGRKRAVAV	99.8
32	QWLVKWVIIKVV	0.5	68	VKRVKQILWRLG	47.3	104	KAVAGARQRWAL	99.8
33	VQRIIWLRVKIV	0.6	69	KRVQAKAWRLQR	49.0	105	AIGAARAWRQWA	99.8
34	QQVKWWLIRWLA	0.9	70	RARQIRWLRKRV	49.8	106	QLARLARVVWGL	99.8
35	RVLIKWKKVIVV	1.0	71	KIQRRAWKQWRK	52.9	107	AVIVRAAKGGAR	99.8

Supplementary Notes

Statistical analysis and data modeling

To obtain binary predictions, the experimental values for the Training Set were considered at varying threshold values of antibiofilm potency ranging from the top 5%, 10% and 20% of the ranked 1018-derived peptides. The dependent variable was then assigned a value of 1 or -1 when a peptide had a greater or lower experimental value than the threshold. Linear Discriminant Analysis was used to identify the classifier functions and statistical analysis was carried out with STATISTICA version 10.0 (StatSoft Inc. Tulsa, OK. USA). The *forward stepwise* and *best subset* methods were employed for the attribute selection. The tolerance parameter was set to 0.01. P(active) and P(inactive) are the probabilities with which equations could classify a compound as active and inactive, respectively. By using the models, any compound could be classified as either active if ΔP% was >0 (whereby $\Delta P\% = [P(\text{Active}) - P(\text{Inactive})] \times 100$); otherwise the compound was deemed inactive. The quality of the models was determined according to Wilks' λ, the square of the Mahalanobis distance D^2, Fisher ratio (F), significance level (p) and the percentage of good classification (accuracy, Q). Therefore, parameters like sensitivity 'hit rate' (SE), specificity (SP), false positive rate (fp_{rate}) and Matthews' correlation coefficient (MCC) were taken into account[1]. Those models with high statistical significance but having the minimal number of molecular descriptors were preferred. We chose the top 5% as optimum cut off value after assessing the accuracy of the models at different cut off values (ranging from 5% to 20%) using all the parameters mentioned above. Then a 10-fold cross-validation was performed on the final set using the top 5% as the optimum threshold value. Briefly, to perform the cross-validation procedure, 10% of the peptides in the Training Set were randomly selected as validation data set while the rest of the peptides were used as a corresponding training set. This was repeated a total of 10 times resulting in 10 validation sets and 10 training sets created.

Virtual set sequence requirements

In general, antibiofilm peptides share sequence similarity with conventional antimicrobial peptides, with both classes being enriched for cationic and hydrophobic residues and having similar patterns of their distribution across the length of the peptide backbone. Therefore, the sequence requirements for the Virtual Set were devised to limit the sequence space of 12-mer peptides based on our accumulated knowledge of the 9 most common amino acids in the most

active antibiofilm peptides[2,3] while providing similar overall peptide characteristics as the 1018-derived Training Set (**Supplementary Table S3**). The Virtual Set was limited to peptides containing 4-9 hydrophobic residues and 2-6 cationic residues. Tryptophan residues are also known to play an important role in membrane association[4], and were favourable substitutions in 8 of 12 amino acid positions of 1018 (**Fig. 1b**) therefore sequences were allowed to contain up to 3 Trp residues. Conversely, only two residues total of Gly and/or Gln were permitted since these are not typically found in traditional antimicrobial peptides and were rarely favoured in our Training Set (**Fig. 1b**). Finally, 1018 aggregation has been observed under specific sample conditions[5] which might limit the therapeutic potential of antibiofilm peptides. Consequently, to prevent potential aggregation, stretches of hydrophobic amino acids were limited to four residues or fewer, which should enable a minimal potential for peptide self-association through hydrophobic surfaces. The peptide sequence requirements of the Virtual Set are summarized in the table below.

Supplementary Table S3 – Peptide sequence constraints used to generate the 100,000 peptide sequences comprising the Virtual Set.

**Characteristic**	**Condition**
Peptide Length	12 Residues
Amino acid composition	G, A, R, K, L, I, V, W, Q
Percent hydrophobic residues	$4 \leq (A+L+I+V+W) \leq 9$ (33-75%)
Percent cationic residues	$2 \leq (R + K) \leq 6$ (17-50%)
Hydrophobic Regions	No more than 4 hydrophobic amino acids together
Tryptophan	$W \leq 3$
Glycine and Glutamine	G or Q or $G+Q \leq 2$
Amino acid diversity	At least 5 different types of amino acids

Supplementary References

1. Baldi, P., Brunak, S., Chauvin, Y., Andersen, C. A. & Nielsen, H. Assessing the accuracy of prediction algorithms for classification: an overview. *Bioinformatics* **16,** 412–424 (2000).
2. de la Fuente-Núñez, C., Reffuveille, F., Haney, E. F., Straus, S. K. & Hancock, R. E. W. Broad-spectrum anti-biofilm peptide that targets a cellular stress response. *PLoS Pathog.* **10,** e1004152 (2014).
3. Haney, E. F., Mansour, S. C., Hilchie, A. L., de la Fuente-Núñez, C. & Hancock, R. E. W. High throughput screening methods for assessing antibiofilm and immunomodulatory activities of synthetic peptides. *Peptides* **71,** 276–285 (2015).
4. Chan, D. I., Prenner, E. J. & Vogel, H. J. Tryptophan- and arginine-rich antimicrobial peptides: Structures and mechanisms of action. *Biochim. Biophys. Acta* **1758,** 1184–1202 (2006).
5. Haney, E. F., Wu, B., Lee, K., Hilchie, A. L. & Hancock, R. E. W. Aggregation and its influence on the immunomodulatory activity of synthetic innate defense regulator peptides. *Cell Chem. Biol.* **24,** 969–980.e4 (2017).
